# Public Pharmacists' Association

**Published:** 1920-12-11

**Authors:** 


### Public Pharmacists' Association.

A meeting of the Public Pharmacists' Association was held at St. Bride Institute, London, E.C., on November 24, 1920. Mr. J. T. P. Hollingworth (Victoria Hospital, Chelsea) presided over a representative gathering of hospital and institutional pharmacists. Several matters of business were dealt with, after which an address was given by Mr. Arthur H. Jenkin (City of London Dispensary) on the "Test Case." The Pharmaceutical Society, which has a Charter and is the examining body for pharmacists, has also been dealing with matters relating to the business aspect of pharmacy. There was an outside proposal to form a separate body to deal purely with "trade" matters as distinct from the professional side. The Pharmaceutical Society argued that they had full powers under their Charter to deal with *all* matters concerning the business of chemist and druggist, and, to settle the matter, it was decided to have a "friendly action" in the courts to get a legal decision. This was designated the "Test Case," and Mr. Jenkin acted as plaintiff with the object of getting an injunction against the Pharmaceutical Society to restrain them from using funds to promote the interests of a section only of its members who keep retail shops. The legal judgment now affirms that the Society cannot act in this nature, and it is likely that its activities will in future be confined to statutory functions—examining, registration, etc.

Mr. Jenkin gave a lucid explanation of the whole subject, and expressed the view that the Pharmaceutical Society would now have more time to devote to the professional side of pharmacy, with which institutional pharmacists are mostly concerned, to raising the status, and preventing the illegal practice of dispensing.

Many questions were asked, to which the speaker replied. The Chairman congratulated Dr. Jas. H. France on his success in obtaining a medical degree, and referred in appreciative terms to the good work which Dr. France had done for the Association as hon. secretary when he was a practising pharmacist. It was decided to elect Dr. France an honorary member of the Association. Miss Job and Mr. Fulleylove were elected members.

